# Preparation, characterisation, and controlled release of sex pheromone-loaded MPEG-PCL diblock copolymer micelles for *Spodoptera litura* (Lepidoptera: Noctuidae)

**DOI:** 10.1371/journal.pone.0203062

**Published:** 2018-09-07

**Authors:** Yixin Chen, Xiuqin Chen, Yong Chen, Hui Wei, Shuo Lin, Houjun Tian, Tao Lin, Jianwei Zhao, Xiaojun Gu

**Affiliations:** 1 Institute of Plant Protection, Fujian Academy of Agricultural Sciences, Fuzhou, Fujian, China; 2 Fujian Key Laboratory for Monitoring and Integrated Management of Crop Pests, Fuzhou, Fujian, China; 3 Fuzhou Scientific Observing and Experimental Station of Crop Pests, Ministry of Agriculture, Fuzhou, Fujian, China; 4 Key Laboratory of Green Control of Insect Pests, Fujian Agriculture and Forestry University, Fuzhou, Fujian, China; 5 College of Plant Protection, Fujian Agriculture and Forestry University, Fuzhou, Fujian, China; Institute for Bioscience and Biotechnology Research, ITALY

## Abstract

Sex pheromones are important for agricultural pest control. The main sex pheromone components of *Spodoptera litura* are (Z,E)-9,11- and (Z,E)-9,12-tetradecadienyl acetate (Z9,E11-14:Ac; Z9,E12-14:Ac). In this study, we investigated the optimal conditions for encapsulation of *S*. *litura* sex pheromonesin micelles via the self-assembly method using monomethoxy poly (ethylene glycol)-poly (ε-caprolactone) (MPEG-PCL) as a biodegradable wall-forming material with low toxicity. In the L_9_(3^4^) orthogonal experiment, 3 amphiphilic block copolymers, with different hydrophilicity to hydrophobicity ratios, were examined. Optimal encapsulation conditions included stirring of MPEG_5000_-PCL_2000_ at 1000 rpm at 30°C with 2.5:1 wall-forming: core material mass ratio. *S*. *litura* sex pheromone-loaded MPEG_5000_-PCL_2000_ micelles presented a homogeneous spherical morphology with apparent core-shell structure. The release kinetics of optimized MPEG_5000_-PCL_2000_ micelles was best explained by a first-order model. Encapsulated Z9,E11-14:Ac and Z9,E12-14:Ac were released slowly, not suddenly. Methyl oleate (MO) was used as an agent to control micellar release performance. When MO content equalled block content, micelle half-life could be prolonged, thereby controlling the release speed. Overall, our results showed MPEG-PCL as a promising controlled-release substrate for sex pheromones.

## Introduction

*Spodoptera litura* Fabricius (Lepidoptera: Noctuidae), a type of polyphagous pest with an aggressive eating pattern, has a wide range of hosts, encompassing approximately 200 kinds of plants [1] including those of various vegetables, fruits, baccies, cotton, corn, tea, and other cash crops [2]. However, due to the overuse of chemical agents to prevent and control this pest, insecticide-resistance of *S*. *litura* has gradually increased [3–6]. The use of synthetic sex pheromone traps to monitor population densities of *S*. *litura* on crops is a simple and practical method applied in many cropping systems [7]. The main components of the sex pheromones of *S*. *litura*, namely, (Z9,E11)-tetradecadienyl acetate (Z9,E11-14:Ac) and (Z9,E12)-tetradecadienyl acetate (Z9,E12-14:Ac), were isolated and identified by Tamaki and colleagues in the early 1970s [8, 9]. In an attempt to replace traditional chemical insecticides, sex pheromones have been employed to control *S*. *litura* in various countries; sex pheromones have also been used to trap [10, 11] or disorient [12–14] pests. Sustained-release materials are an important part of the medium in which the pheromones are constituted and influence the effects and duration of release of the pheromones. At present, insect sex pheromone-releasing dispensers mainly consist of rubber septa heads, plastic tubes, hollow fibres, and paraffin oil [15–17].

Nanotechnology has enormous potential in various fields, used in the development of insecticides, pharmaceuticals [18,19], and electronics. In addition, in the field of agriculture, nanomaterials have been developed for the management of insect pests [20, 21]. Encapsulation of hydrophobic pheromones into nanoparticles is an advanced and effective application method. Recently, the use of microcapsules has also received increased attention [22, 23]. Microcapsulation can reduce the influence of the surrounding environment on encapsulated materials, prolong the duration of activity of the compounds, or reduce their toxicity, and avoid contamination of the effective ingredients by preventing mixture with other substances. The use of sex pheromone microcapsules in sustained-release dosage forms to disorient and control pests, such as *Plutellaxy lostella* (L.), *Cydia pomonella* (L.), *Tuta absoluta* (Meyrick), *Grapholita molesta* (Busck), *Argyrotaenia velutinana* (Walker), *Choristoneura rosaceana* (Harris), *Pectinophora gossypiella* (Saunders), and *Keiferia lycopersicella* (Walsingham) has been reported in various countries [13, 24–28].

Monomethoxy poly (ethyleneglycol)-poly (ε-caprolactone) (MPEG-PCL) has been widely studied due to its biocompatibility, biodegradability, easy production, non-toxicity, and thermo-sensitivity that promise its widespread applications [29–32]. Polymeric micelles are more stable than surfactant micelles and have lower critical micelle concentrations and degradation rates [33]. MPEG-PCL copolymers that self-assemble into nanoparticles with the core-shell structure include a hydrophobic PCL core and a hydrophilic MPEG shell. During the process of self-assembly, agents can be effectively loaded into the hydrophobic nucleus through physical packaging [34], which allows the agent to retain its activity, decreases its volatilisation, and permits sustained release [35, 36]. Thus, polymeric micelles can be a versatile system for the effective delivery of different classes of agents.

In this study, three kinds of MPEG-PCL, with different proportions of hydrophobicity and hydrophilicity, were selected as wall-forming materials for incorporation into *S*. *litura* sex pheromone containing micelles formed by amphiphilic block copolymer self-assembly, and the optimal experimental conditions and pheromone release performance were evaluated to examine the effect of controlled-release agent incorporation during micelle assembly.

## Materials and methods

### Materials

Z9,E11-14:Ac (97%) and Z9-E12-14:Ac (97%) were purchased from Sigma-Aldrich Inc. (St. Louis, MO, USA); MPEG_5000_-PCL_2000_, MPEG_3000_-PCL_2300_, and MPEG_5000_-PCL_10000_ were purchased from Jinan Daigang Bio-engineering Co., Ltd. (Jinan, China); N, N—dimethylformamide (DMF, analytical grade) and Tween-20 (analytical grade) were from Sinopharm Chemical Reagent Co., Ltd. (Shanghai, China); methyl oleate (MO, chemically pure) was from Macklin Reagent Co., Ltd. (Shanghai, China).

### Critical micelle concentration

The critical micelle concentration (CMC) of MPEG-PCL was measured by a UV spectrophotometer (TU-1810, Beijing Purkinje General Instrument Co., Ltd.). Three kinds of MPEG-PCL, with different proportions of hydrophobic and hydrophilic components were dissolved in deionized water to obtain a stock solution of concentration 1.000 g/L. The absorption maxima of the different concentrations of MPEG-PCL (0.001, 0.005, 0.008, 0.01, 0.04, 0.05, 0.1, 0.2, 0.35, 0.5, 0.7, and 1 g/L) was recorded and mapped with the values of lgA-lgC (A: absorption, C: concentration); critical micelle concentration of block copolymer corresponded to the concentration at which the first derivative curve reached zero [37].

### Preparation of micelles

MPEG-PCL micelles were prepared by the self-assembly method [38]. Briefly, desired amounts of MPEG-PCL and *S*. *litura* sex pheromones (Z9,E11-14:Ac: Z9,E12-14:Ac = 9:1) were individually dissolved in 1 mL DMF and mixed well by adding Tween-20 [0.01% (v/v)]. At a fixed temperature and stirring speed (RH D W S25 stirrer, IKA Company, Germany) (Table 1), 8 mL deionised water was added drop-wise into the solution. The micelle solution was stirred for 30 min, dialysed using dialysis tubing (MD44-3.5, Viskase Co., Lombard, IL, USA) for 24 h, accompanied by stirring. The resulting micellar solution was filtered using a syringe filter (pore size: 0.22 m) to remove aggregates.

**Table 1:** Experimental factors and their levels in orthogonal projects.

Level	W (A)	W/S ratio (w/w) (B)	T (°C) (C)	S (rpm) (D)
**1**	MPEG_5000_-PCL_2000_	2:1	30	800
**2**	MPEG_3000_-PCL_2300_	2.5:1	40	1000
**3**	MPEG_5000_-PCL_10000_	5:1	50	1200

W (wall-forming materials), W/S ratio (the mass ratio of sex pheromone to wall-forming materials), T (reaction temperature), S (stirring speed).

### Orthogonal experimental design

An L_9_ (3^4^) orthogonal table (Table 1) was adopted for this test. The investigated factors included wall-forming materials (W), mass ratio of sex pheromone to wall-forming materials (W/S ratios), reaction temperature (T), and stirring speed (S); encapsulation efficiency of the micelles (EE) was considered as the assessment index. The optimised formulation was prepared in triplicate.

### Determination of entrapment efficiency

Briefly, 0.5 mL of the sex pheromone-loaded micelle solution was fully mixed with 0.5 mL ultrapure water. The solution was extracted with 1 mL n-hexane and completely disrupted using an ultrasound sonicator (Scientz-IID, Ningbo Scientz Biotechnology Co., China) on ice. The encapsulated sex pheromone was dissolved in the hexane after 30 min. The concentration of sex pheromone was determined by gas chromatography (GC, Agilent 7890B, Agilent Technologies, Santa Clara, CA, USA). For GC, a capillary column (HP-5, 30 m × 0.32 mm × 0.25 μm) with a flame ionisation detector and a splitless injector, with nitrogen as the carrier gas, was used. GC conditions were as follows: the column temperature set at 80°C (held for 5 min), raised to 210°C at 10°C/min, and held at 210°C for 15 min.

A standard curve was generated according to the concentration of sex pheromones and peak area; quantity of each component in the sex pheromone was determined from the standard curve. The standard curve regression equations of Z9,E11-14:Ac and Z9,E12-14:Ac were y = 16806x + 50.5 (R^2^ = 0.9997) and y = 18672x − 3.4706 (R^2^ = 0.9999). The sex pheromone entrapment efficiency was calculated using Eq 1:

$$\text{EE}(\%)=\frac{\text{Sex}\;\text{pheromone}\;\text{entrapped}\;\text{in}\;\text{micells}}{\text{Theory}\;\text{sex}\;\text{pheromone}\;\text{loading}\;}\times 100$$ (1)

### Characterisation of micelles

#### Particle morphology

The micelle morphology was observed by transmission electron microscope (TEM, HT 7700, Hitachi, Tokyo, Japan), and the speeding voltage during the test was 80 kV. Samples were prepared by dropping the micelle solution on a carbon-coated copper net, followed by air drying, and dyeing with 0.2 wt% phosphotungstic acid.

#### Determination of particle size

The particle size and its distribution were analysed using a Malvern nanometre particle size analyser (MNPSA) (Zetasizer Nano S90, Malvern Instruments Ltd., Malvern, UK).

#### Stability of micelles

Micelles were stored at 2, 4, and 8°C in the dark. In order to evaluate the physical stability of nanoparticles during this storage period, particle size distribution was monitored at time intervals of 0, 15, and 30 days, using the method described in the section “Determination of particle size”.

### Release performance

#### Sex pheromone release

To evaluate sex pheromone release, the micelles were transferred to a centrifuge tube and placed in an artificial climate chamber (MGC-450HP2, Shanghai Yiheng Co., China) with controlled temperature in the range of 35 ± 3°C, light: dark cycle of 12 h:12 h, and relative humidity of 75 ± 5% for a period of 28 days. The samples were taken out of the artificial climate chamber at regular time intervals for sex pheromone examination by GC. To evaluate the release of sex pheromones from micelles prepared under optimal conditions, the samples were examined every day during the first 14 days, and every 7 days during the subsequent 14 days. To evaluate the release from micelles containing the controlled-release agent, the samples were examined every 3 days over a period of 15 days. Three samples were used in each experiment.

Sex pheromone release was expressed as percentage of accumulated release, since this enabled the evaluation of performance of different micelles. Accumulated release was calculated using Eq 2:

$$\text{Accumulated}\;\text{release}\;(\%)=\frac{{\text{W}}_{0}-{\text{W}}_{\text{t}}}{{\text{W}}_{0}}\times 100$$ (2)

where W_0_ is the sex pheromone content at the initial time and W_t_ is the sex pheromone content at each recorded time.

#### Sex pheromone release kinetics for optimized micelles

For a better understanding of the efficacy of sex pheromones, their release kinetics were studied. Selection of a suitable kinetic model for fitting the sex pheromone release data helped determine the release characteristics. There are a number of kinetic models that describe the overall release of sex pheromone from the vehicle. The most common mathematical models used are: zero-order model (Eq 3), first-order model (Eq 4), Higuchi model (Eq 5), Korsmeyer-Peppasmodel (Eq 6), and Hixson-Crowell model (Eq 7) [39–45]:

$${\text{C}}_{\text{t}}{=\text{C}}_{0}+{\text{K}}_{0}\cdot \text{t}$$ (3)

$${\text{lnC}}_{\text{t}}{=\text{lnC}}_{0}+{\text{K}}_{1}\cdot \text{t}$$ (4)

$${\text{C}}_{\text{t}}{=\;\text{C}}_{0}+{\text{K}}_{\text{H}}\cdot {\text{t}}^{1/2}$$ (5)

$${\text{C}}_{\text{t}}{=\;\text{C}}_{0}+{\text{K}}_{\text{kP}}\cdot {\text{t}}^{\text{n}}$$ (6)

$${\text{C}}_{0}{}^{1/3}{-\text{C}}_{\text{t}}{}^{1/3}={\text{K}}_{\text{HC}}\cdot \text{t}$$ (7)

where

C_t_—amount of drug released in time t,

C0—the initial amount of drug,

K0—zero-order kinetic constant,

K1—first-order kinetic constant,

K_H_—Higuchi kinetic constant,

K_KP_—Korsmeyer-Peppas release constant,

KHC—Hixson-Crowell release constant,

n—diffusional release exponent,

t—time.

#### Half-life calculations

Depletion of pheromone components from the micelle formulations was characterised by the first-order kinetic model: lnC_t_ = lnC_0_ +K_1_·t. Half-lives (t_1/2_) for compounds were determined from the exponential equation, substituting calculated values of C_0_ and K_1_, and setting (C_t_/C_0_) to 0.5 [46].

#### Statistical analysis

Statistical analysis was done with SPSS 17.0 software package (Chicago, IL, USA). One-way analysis of variance (ANOVA) for independent samples followed by Duncan’s multiple range tests were performed to evaluate the quantitative results. Data were obtained from triplicate samples and, expressed as mean ± standard error (SE); values of P ≤0.05 and P ≤ 0.01 were considered statistically significant and extremely significant, respectively.

## Results and discussion

### Critical micelle concentration

The CMC of MPEG_5000_-PCL_2000_, MPEG_3000_-PCL_2300_, and MPEG_5000_-PCL_10000_ were 0.011 g/L, 0.00199g/L, and0.00102g/L, respectively. The low CMC may better stabilise the micelles in suspension.

### Optimisation of MPEG-PCL micelle formation

The results of the L_9_(3^4^) orthogonal experiments using MPEG-PCL nanoparticles are shown in Tables 2 and 3. The range values (R) of the factors indicated their effects on the efficiency of encapsulation of Z9,E11-14:Ac in the order: S (D) > T (C) > W (A) > W/S ratios (B), and that of Z9,E12-14:Ac in the order: S (D) > W (A) > T (C) > W/S ratios (B). The K value showed the optimal encapsulation group to be W (MPEG_5000_-PCL_2000_)—W/S ratios (2.5:1)—T (30°C)—S (1000 rpm) for both Z9,E11-14:Ac and Z9,E12-14:Ac.

**Table 2:** Results of the L_9_(3^4^) orthogonal experiment using Z9,E11-14:Ac MPEG-PCL nanoparticles.

Factor	W (A)	W/S ratio (w/w) (B)	T (°C) (C)	S (rpm) (D)	EE (%) (Z9:E11-14:Ac)%
**1**	1	1	1	1	76.76
**2**	1	2	2	2	76.51
**3**	1	3	3	3	51.10
**4**	2	1	2	3	53.95
**5**	2	2	3	1	65.45
**6**	2	3	1	2	78.04
**7**	3	1	3	2	61.26
**8**	3	2	1	3	55.77
**9**	3	3	2	1	60.74
**K1**	204.37	191.96	210.57	202.95	
**K2**	197.44	197.73	191.20	215.81	
**K3**	177.77	189.89	177.80	160.82	
**k1**	68.12	63.99	70.19	67.65	
**k2**	65.81	65.91	63.73	71.94	
**k3**	59.26	63.30	59.27	53.61	
**R**	8.87	2.61	10.92	18.33	
**Influence degree of factors**	S> T> W> W/S ratios				
**Best group**	A_1_B_2_C_1_D_2_ W (MPEG_5000_-PCL_2000_)—W/S ratio (2.5:1)—T (30°C)—S (1000 rpm)				

W (wall-forming materials), W/S ratio (the mass ratio of sex pheromone to wall-forming materials), T (reaction temperature), S (stirring speed), EE (encapsulation efficiency of micelle). The arrangements of A, B, C, D were decided by orthogonal design for 4 (factor) × 9 (run number).

**Table 3:** Results of the L_9_(3^4^) orthogonal experiment using Z9,E12-14:Ac MPEG-PCL nanoparticles.

Factor	W (A)	W/S ratio (w/w) (B)	T (°C) (C)	S (rpm) (D)	EE (%) (Z9:E12-14:Ac)%
**1**	1	1	1	1	77.11
**2**	1	2	2	2	85.77
**3**	1	3	3	3	58.91
**4**	2	1	2	3	55.41
**5**	2	2	3	1	71.66
**6**	2	3	1	2	82.73
**7**	3	1	3	2	60.23
**8**	3	2	1	3	56.45
**9**	3	3	2	1	57.95
**K1**	221.79	192.74	216.29	206.72	
**K2**	209.79	213.88	199.13	228.73	
**K3**	174.63	199.59	190.80	170.77	
**k1**	73.93	64.25	72.10	68.91	
**k2**	69.93	71.29	66.38	76.24	
**k3**	58.21	66.53	63.60	56.92	
**R**	15.72	7.05	8.50	19.32	
**Influence degree of factors**	S> W> T> W/S ratios				
**Best group**	A_1_B_2_C_1_D_2_ W (MPEG_5000_-PCL_2000_)—W/S ratio (2.5:1)—T (30°C)—S (1000 rpm)				

W (wall-forming materials), W/S ratio (the mass ratio of sex pheromone to wall-forming materials), T (reaction temperature), S (stirring speed), and EE (encapsulation efficiency of micelle). The arrangements of A, B, C, D were decided by orthogonal design for 4 (factor) × 9 (run number).

Conversely, it was much easier to draw a more intuitive conclusion from the results by range analysis of the orthogonal experiment. However, the calculation processes were extensive and could not evaluate the errors; thus, it was necessary to carry out variance analysis of the orthogonal experiment results. It can be seen from the variance analysis tables (Tables 4 and 5) that except for the W/S ratios, all the other factors (including W, S, and T) had significant effects on the experimental results. The order of factors affecting the encapsulation efficiency of Z9,E11-14:Ac and Z9,E12-14:Ac, obtained from variance analysis, was the same as that from the range analysis.

**Table 4:** Analysis of the orthogonal experiment results of Z9,E11-14:Ac MPEG-PCL nanoparticles using ANOVA.

Source of variation	*SS*	*Df*	*MS*	F
**A**	380.931	2	190.466	7.554**
**B**	33.001	2	16.501	6.540*
**C**	542.744	2	271.372	10.763**
**D**	1655.042	2	827.521	32.821**
**error**	453.842	18	25.213	

*SS* (Sum of square), *df* (degree of freedom), *MS* (mean square), F (critical value).

‘*’and ‘**’represent significant difference (*P* ≤ 0.05) and extremely significant difference (*P* ≤ 0.01), respectively.

**Table 5:** Analysis of the orthogonal experiment results of Z9,E12-14:Ac MPEG-PCL nanoparticles using ANOVA.

Source of variation	*SS*	*Df*	*MS*	F
**A**	121.833	2	600.917	20.087**
**B**	232.648	2	116.324	3.888*
**C**	337.751	2	168.875	5.645*
**D**	1711.861	2	855.930	28.611**
**Consolidated error**	538.493	18	29.916	

*SS* (Sum of square), *df* (degree of freedom), *MS* (mean square), F (critical value).

‘*’and ‘**’represent significant difference (*P* ≤ 0.05) and extremely significant difference (*P* ≤ 0.01), respectively.

Encapsulation efficiency of the micelles was controlled by the length of hydrophobic or hydrophilic chain (wall-forming materials), W/S ratio, T, and S. Based on the two analyses, it was concluded that the order of effect of W and T on the encapsulation efficiency was different. Since the mass ratio of Z9,E11-14:Ac was much larger than that of Z9,E12-14:Ac, factor S was regarded as the most important factor affecting the encapsulation efficiency followed by T, W, and W/S ratios. S likely played an important role in the formation of micelles, since the sex pheromone should be well mixed in the process of micelle formation, and a certain speed would be required when water is added to the solution to conjugate the hydrophilic ends of the amphiphilic block copolymer. The influence of W was determined by the length of hydrophobic and hydrophilic chains, whereas T likely influenced micellar assembly and speed of sex pheromone volatility to lessen the encapsulation efficiency. However, the influence of W/S ratios on encapsulation efficiency was relatively small.

The optimal conditions for *S*. *litura* sex pheromone encapsulation with MPEG-PCL, determined from the above results, involved stirring MPEG_5000_-PCL_2000_ at a speed of 1000 rpm at 30°C with a 2.5:1 mass ratio of wall-forming to core materials. Based on these conditions, three parallel experiments with MPEG-PCL micelles were subsequently conducted (Table 6). The results consistently showed that entrapment efficiency was the highest among the combinations used in the orthogonal experiments, which verified the utility and feasibility of the conditions.

**Table 6:** Test of verification.

Best group	EE (%)	
	Z9,E11-14:Ac	Z9,E12-14:Ac
**A**_**1**_**B**_**2**_**C**_**1**_**D**_**2**_	82.91	88.73
**A**_**1**_**B**_**2**_**C**_**1**_**D**_**2**_	84.54	87.84
**A**_**1**_**B**_**2**_**C**_**1**_**D**_**2**_	82.21	89.32
**Mean ± SE**	83.22 ± 0.35	88.63 ± 0.56

EE (encapsulation efficiency of micelle), A_1_B_2_C_1_D_2_ [best group: W (MPEG_5000_-PCL_2000_)—W/S ratio (2.5:1)—T (30°C)—S (1000 rpm)]

### Characterisation of microcapsules

**Fig 1:**
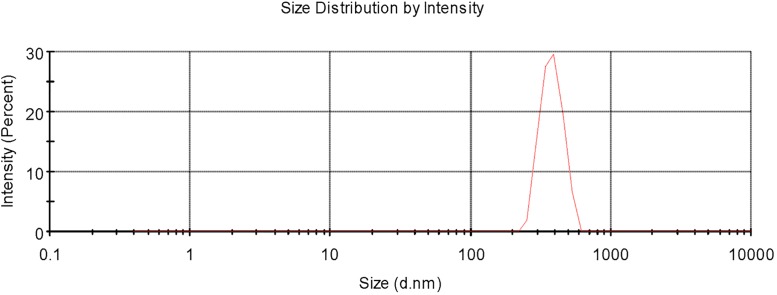
Particle size distribution of sex pheromone-loaded MPEG_5000_-PCL_2000_ nanoparticles.

**Fig 2:**
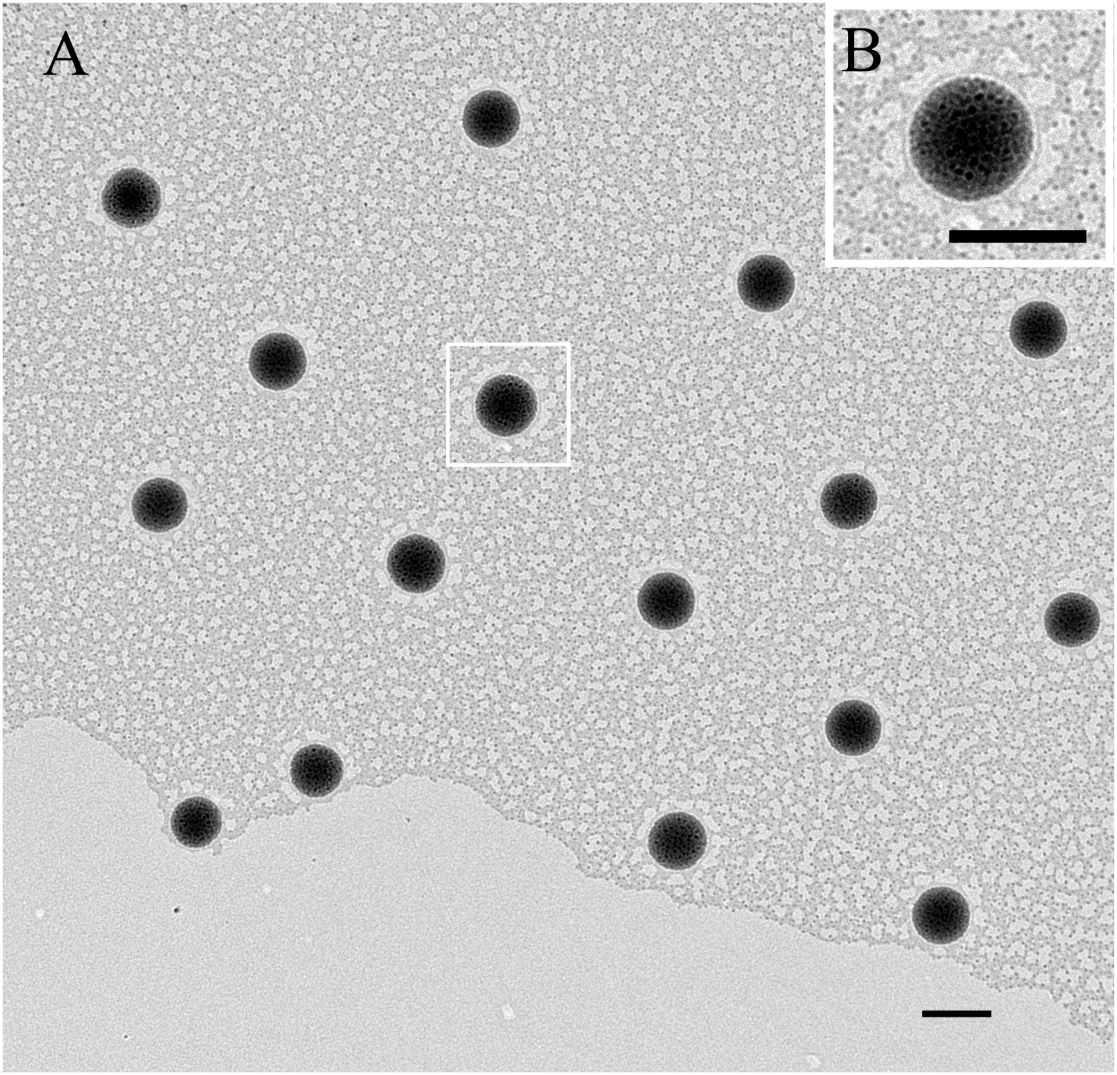
TEM micrographs of sex pheromone-loaded MPEG_5000_-PCL_2000_ nanoparticles. Bars in panel A and panel B are 400 nm. Panel B is an enlarged view of the boxed area in Panel A.

For fresh MPEG_5000_-PCL_2000_ nanoparticles, prepared according to the optimised formulation and preparation conditions, the particle size was 374 ± 5.13 nm by MNPSA (Fig 1). The formation of micellar nanostructures was confirmed by TEM. The MPEG_5000_-PCL_2000_ nanoparticles showed a homogeneous spherical morphology, with average diameter of 300 nm, presenting an apparent core-shell structure (Fig 2). The size of the MPEG_5000_-PCL_2000_ nanoparticles, measured by TEM, was smaller compared to that from MNPSA measurements, since the former was related to the collapsed nanoparticles after water evaporation, whereas the latter represented their hydrodynamic diameter [47].

After preparation, the micelles were dispersed in aqueous medium. Therefore, stability of their sizes was of great importance, both as a measure of particle structure integrity and as an indicator of the possible inter-particular associations (aggregation). At sub-zero temperatures, the solution solidified and the micellar structure lost its integrity. For this purpose, we chose 2, 4, and 8°C as the storage temperatures, at which the particle size was monitored in the dark over a period of 30 days. The variation of micellar size as a function of storage time is shown in Table 7. All the micelles increased slightly in size, throughout the measurement period, at different temperatures. This observation could not be an indicator of aggregation, which usually leads to a several-fold increase in size; instead, copolymer swelling and/or hydration may be responsible for this event [48]. Since the variation of micellar size was less when stored at 2°C, we chose to store the micelles at 2°C in the dark for the best storage conditions.

**Table 7:** Stability of micelles during storage period.

Store temperature (°C)	Mean size of micelles immediately after preparation (nm)	Mean size of micelles after 15 day (nm)	Mean size of micelles after 30 day (nm)
2	374	377	386
4	374	379	391
8	374	384	402

### Sex pheromone release kinetics in optimized micelles

The sex pheromone release results of MPEG_5000_-PCL_2000_ micelles were used in various mathematical models to evaluate the kinetics and mechanism of release from the micelles. Based on the correlation coefficient (R) value in various models, the one that fit best with the release data was selected; the one with a high ‘R’ value was considered as the best fit. The release constant was calculated from the slopes of the appropriate models, and the regression coefficient (R^2^) was determined (Table 8).

**Table 8:** Results of release kinetics model fitting of MPEG_5000_-PCL_2000_ micelles.

Model	Z9,E11-14:Ac			Z9,E12-14:Ac		
	Intercept	Slope	R^2^	Intercept	Slope	R^2^
**Zero-order model**	25.5268	3.2234	0.8599	22.2329	3.1157	0.8670
**First-order model**	0.2101	0.0853	0.9598	0.1886	0.0721	0.9608
**Higuchi model**	1.0032	-0.2014	0.9364	1.0237	-0.1932	0.9398
**Korsmeyer-Peppas model**	0.1221	0.69(n)	0.9298	0.0952	0.58(n)	0.9339
**Hixson-Crowell model**	0.4441	-0.0230	0.6861	0.4736	-0.0236	0.6987

According to the results shown in Table 8, the profile of Z9,E11-14:Ac and Z9,E12-14:Ac release from micelles fit best to the first-order kinetic model where the highest linearity was achieved (Z9,E11-14:Ac R^2^ = 0.9598; Z9,E12-14:Ac R^2^ = 0.9608). Therefore, the sex pheromone release mechanism was assumed to be diffusion-controlled. When analysed according to the Korsmeyer-Peppas model, the diffusion exponent was found between 0.5 and 1.0 (Z9,E11-14:Ac n = 0.69, Z9,E12-14:Ac n = 0.58), based on which the diffusional release was assumed to follow anomalous transport.

### Release performance of optimized micelles

**Fig 3:**
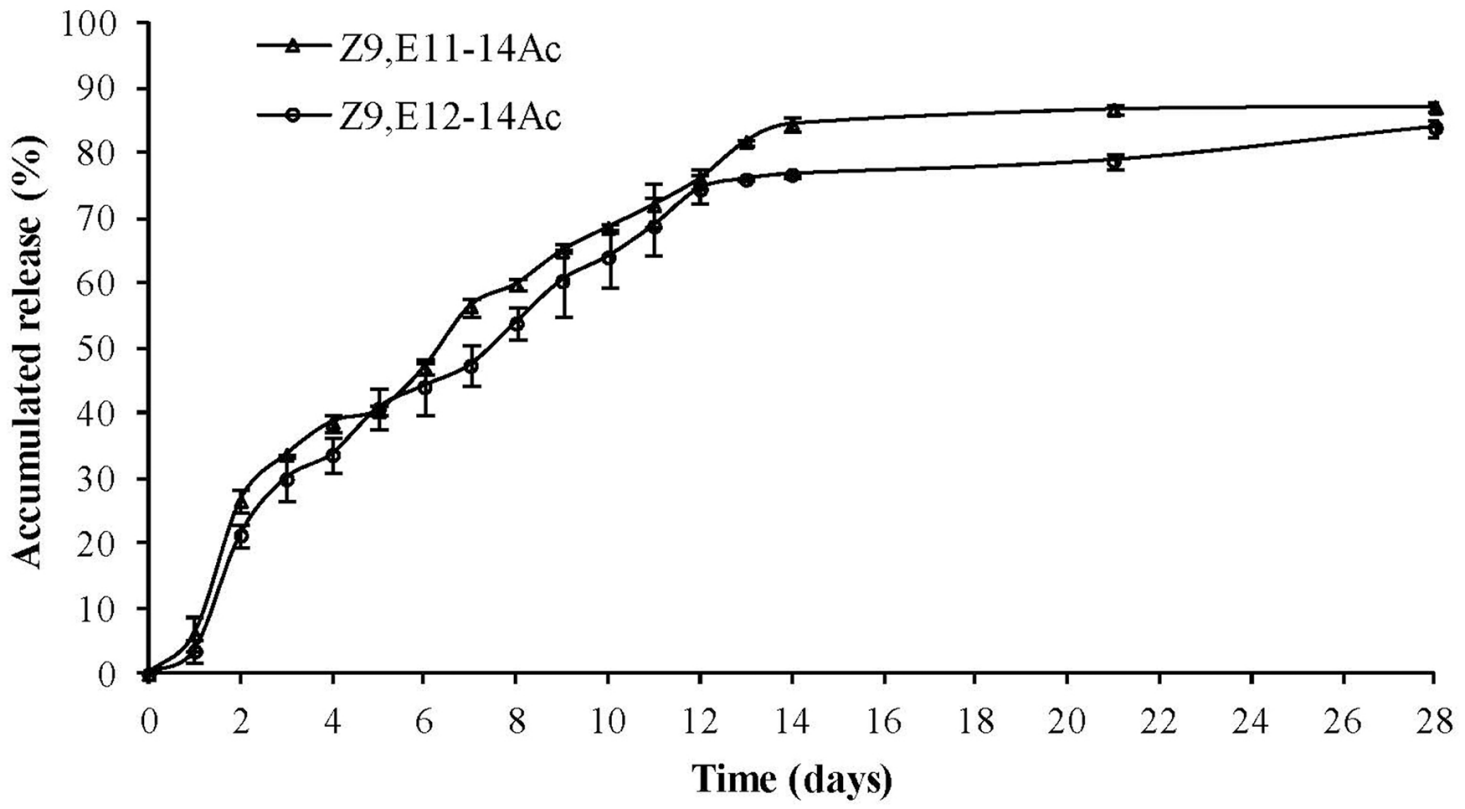
Plot of accumulated release with time in case of sex pheromone-loaded MPEG_5000_-PCL_2000_ micelles. The *S*. *litura* sex pheromone has two components, Z9,E11-14:Ac and Z9,E12-14:Ac. Data are expressed as means ± S.D. (n = 3).

The plot of accumulated release from sex pheromone-loaded MPEG_5000_-PCL_2000_ micelles indicated that Z9,E11-14:Ac could be released from micelles faster than Z9,E12-14:Ac, in a sustained manner. The two components had a high release rate in the first 3 days, which was attributed to the fact that nanoparticles usually contain sex pheromone not only at the inner core but also on their surface. After this initial loss, sex pheromone release approximated first-order release rates more closely [49]. Accordingly, following the first burst release period, sex pheromone was released slowly, independent of the initial sex pheromone concentration in the micelles. As shown in Fig 3, from day 4 to 14, the release rate tended to slow down and remained constant. After 14 days, the release rate decreased further and tended to be stable, although the release rate of Z9,E11-14:Ac was less than that of Z9,E12-14:Ac. According to the first-order kinetic model, the half-life of Z9,E11-14:Ac and Z9,E12-14:Ac in the micelle was 5.6 and 7.0 days, respectively. The half-life difference of 1.4 days may have been due to the different proportions of sex pheromone components in the micelle. Based on the results of this study, we found that Z9,E11-14:Ac and Z9,E12-14:Ac were released slowly from MPEG_5000_-PCL_2000_ micelles, and that no sudden release occurred throughout the process, thereby indicating that diblock copolymer micelles were suitable for use as a controlled substrate. Our studies indicated that although MPEG-PCL diblock copolymer micelles did not maintain a constant release rate, they met the first-order kinetic model requirements, with adynamic rapid-to-slow release, lasting for almost a month. Other release carriers, such as PVC, have demonstrated equal or better release duration for that pheromone [46]. However, the micelles in this study were in aqueous solution and hence environment friendly; they were physically and chemically stable, non-toxic, and biodegradable.

### Influence of a controlled-release agent on the controlled release performance of micelles

**Fig 4:**
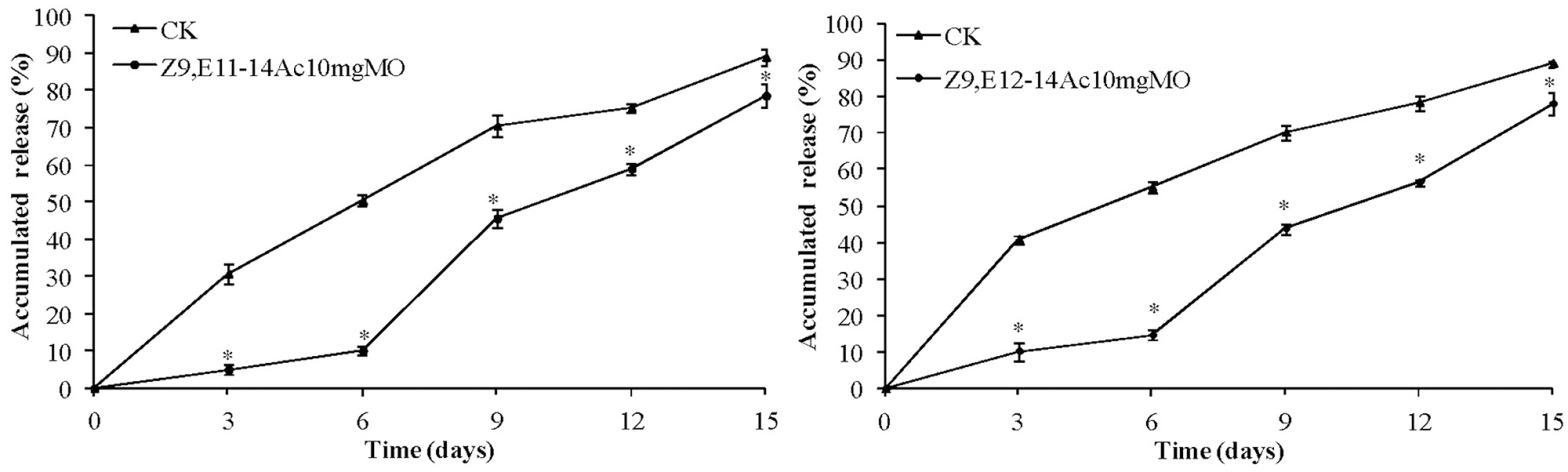
Accumulated release of sex pheromone-loaded MPEG_5000_-PCL_2000_ micelles containing controlled-release agents (A) Z9,E11-14:Ac and (B) Z9,E12-14:Ac. Data are expressed as means ± S.D. (n = 3). Asterisks (*) indicate significance (P≤ 0.05).

Table 9 shows that while the differences among the tested concentrations of MO were not significant, when the mass of wall-forming materials equalled that of MO (10 mg/mL), the half-life of Z9,E11-14:Ac and Z9,E12-14:Ac in the micelle increased by 3.7 and 4.2 days, respectively, compared to that of the control. With the increased content of controlled-release agent, the efficiency of controlled release declined, potentially due to the organic liquid which may have affected micelle formation and inhibited the encapsulation efficiency, thereby impacting the release rate. Fig 4 shows that MO, as a controlled-release agent, could retard the overall release rate of micelles, especially in the first 3 days without burst release. Compared to that of the control, release rate of the two components was slower over the first 6 days. From day 7 to 15, the release rates increased relative to that during the first 6 days. Thus, addition of appropriate quantities of MO into the micelle could prolong the half-life and control the release performance.

**Table 9:** Influence of a controlled-release agent on the controlled-release performance of sex pheromone-loaded MPEG_5000_-PCL_2000_ micelles.

Compound	Mass of MO (mg/mL)	Half-life (days)	First-order release parameters^a^		
			Slope	Intercept	R^2^
**Z9,E11-14:Ac**	CK	5.4	-0.1395	0.0636	0.9866
	10	9.1	-0.1021	0.2326	0.9432
	20	8.7	-0.1064	0.2284	0.9485
	30	8.6	-0.1086	0.2377	0.9475
**Z9,E12-14:Ac**	CK	5.0	-0.1374	-0.0127	0.9919
	10	9.2	-0.0967	0.1935	0.9432
	20	8.9	-0.0998	0.1988	0.9435
	30	8.7	-0.1032	0.2064	0.9428

^a^Thefirst-order kinetic model: lnC_t_ = lnC_0_ +K_1_·t, R^2^ = regression coefficient.

## Conclusions

The optimal preparation conditions of *S*. *litura* sex pheromone-amphiphilic block copolymer micelles were shown to involve stirring MPEG_5000_-PCL_2000_ at a speed of 1000 rpm at 30°C with a 2.5:1 mass ratio of wall-forming to core materials. The nanoparticles presented a homogeneous spherical morphology with an apparent core-shell structure, and were free from the inter-micellar adhesion phenomena. The release kinetics of optimized MPEG_5000_-PCL_2000_ micelles was best explained by first-order model. Since the release from micelles was slow, without a sudden-release phenomenon, the amphiphilic copolymer was considered suitable for use as a controlled substrate. When the mass of added MO equalled that of wall-forming materials, the half-life could be prolonged, thereby allowing control of the release rate. These results indicated that the diblock copolymer could be a suitable controlled-release substrate, and the micelles could have potential use in the control applications of mass trapping and mating disruption in the field.

## Supporting information

S1 Fig Particle size distribution of sex pheromone-loaded MPEG_5000_-PCL_2000_ nanoparticles. (TIF)

S2 Fig TEM micrographs of sex pheromone-loaded MPEG_5000_-PCL_2000_ nanoparticles. Bars in panel A and panel B are 400 nm. Panel B is an enlarged view of the boxed area in Panel A.
(TIF)

S3 Fig Plot of accumulated release with time in case of sex pheromone-loaded MPEG_5000_-PCL_2000_ micelles. The *S*. *litura* sex pheromone has two components, Z9,E11-14:Ac and Z9,E12-14:Ac. Data are expressed as means ± S.D. (n = 3).
(TIF)

S4 Fig Accumulated release of sex pheromone-loaded MPEG_5000_-PCL_2000_ micelles containing controlled-release agents (A) Z9,E11-14:Ac and (B) Z9,E12-14:Ac. Data are expressed as means ± S.D. (n = 3). Asterisks (*) indicate significance (P ≤ 0.05).
(TIF)

S1 Table Experimental factors and their levels in orthogonal projects. W (wall-forming materials), W/S ratio (the mass ratio of sex pheromone to wall-forming materials), T (reaction temperature), S (stirring speed).
(DOC)

S2 Table Results of the L_9_ (3^4^) orthogonal experiment using Z9,E11-14:Ac MPEG-PCL nanoparticles. W (wall-forming materials), W/S ratio (the mass ratio of sex pheromone to wall-forming materials), T (reaction temperature), S (stirring speed), EE (encapsulation efficiency of micelle). The arrangements of A, B, C, D were decided by orthogonal design for 4 (factor) × 9 (run number).
(DOC)

S3 Table Results of the L_9_ (3^4^) orthogonal experiment using Z9,E12-14:Ac MPEG-PCL nanoparticles. W (wall-forming materials), W/S ratio (the mass ratio of sex pheromone to wall-forming materials), T (reaction temperature), S (stirring speed), and EE (encapsulation efficiency of micelle). The arrangements of A, B, C, D were decided by orthogonal design for 4 (factor) × 9 (run number).
(DOC)

S4 Table Analysis of the orthogonal experiment results of Z9,E11-14:Ac MPEG-PCL nanoparticles using ANOVA. *SS* (Sum of square), *df* (degree of freedom), *MS* (mean square), F (critical value). ‘*’and ‘**’ represent significant difference (*P* ≤ 0.05) and extremely significant difference (*P* ≤ 0.01), respectively.
(DOC)

S5 Table Analysis of the orthogonal experiment results of Z9,E12-14:Ac MPEG-PCL nanoparticles using ANOVA. *SS* (Sum of square), *df* (degree of freedom), *MS* (mean square), F (critical value). ‘*’and ‘**’ represent significant difference (*P* ≤ 0.05) and extremely significant difference (*P* ≤ 0.01), respectively.
(DOC)

S6 Table Test of verification. EE (encapsulation efficiency of micelle), A_1_B_2_C_1_D_2_ [best group: W (MPEG_5000_-PCL_2000_)—W/S ratio (2.5:1)—T (30°C)—S (1000 rpm)].
(DOC)

S7 Table Stability of micelles during storage period. (DOC)

S8 Table Results of release kinetics model fitting of MPEG_5000_-PCL_2000_ micelles. (DOC)

S9 Table Influence of a controlled-release agent on the controlled-release performance of sex pheromone-loaded MPEG_5000_-PCL_2000_ micelles. The first-order kinetic model: lnC_t_ = lnC_0_ +K_1_·t, R^2^ = regression coefficient.
(DOC)

## References

[1] Wei HY, Huang YP, Du JW. Sex pheromones and reproductive behavior of *Spodoptera litura* (Fabricius) moths reared from larvae treated with four insecticides. Journal of Chemical Ecology. 2004; 30: 1457–66. 10.1023/B:JOEC.0000037751.86203.10 15503531

[2] Ahmad M, Ghaffar A, Rafiq M. Host plants of leaf worm, *Spodoptera litura* (Fabricius) (Lepidoptera: Noctuidae) in Pakistan. Asian Journal of Agriculture and Biology. 2013; 1: 23–28.

[3] Armes NJ, Wightman JA, Jadhav DR, Ranga Rao GV. Status of insecticide resistance in *Spodoptera litura* in Andhra Pradesh, India. Pest Management Science. 1997; 50: 240–48. 10.1002/(SICI)1096-9063(199707)50:3<240::AID-PS579>3.0.CO;2-9

[4] Shad SA, Sayyed AH, Saleem MA. Cross resistance, mode of inheritance and stability of resistance to emamectin in *Spodoptera litura* (Lepidoptera: Noctuidae). Pest Management Science. 2010; 66: 839–846. 10.1002/ps.1950 20603880

[5] Su J, Lai T, Li J. Susceptibility of field populations of *Spodoptera litura* (Fabricius) (Lepidoptera: Noctuidae) in China to chlorantraniliprole and the activities of detoxification enzymes. Crop Protection. 2012; 42: 217–222. 10.1016/j.cropro.2012.06.012

[6] Abbas N, Shad SA, Razaq M, Waheed A, Aslam M. Resistance of *Spodoptera litura* (Lepidoptera: Noctuidae) to profenofos: Relative fitness and cross resistance. Crop Protection. 2014; 58: 49–54. 10.1016/j.cropro.2014.01.002

[7] Shih CJ, Chu YI. A comparison of the catches of males of *Spodoptera litura* in sex pheromone and light traps and estimates of the capture efficacy of the sex pheromone trap. Plant Protection Bulletin Taipei. 1995; 37:311–17.

[8] Tamaki Y, Noguchi H, Yushima T. Sex pheromone of *Spodoptera litura* (F.) (Lepidoptera: Noctuidae): Isolation, identification, and synthesis. Applied Entomology and Zoology. 1973; 8(3): 200–203. 10.1303/aez.8.200

[9] Tamaki Y, Yushima T. Sex pheromone of the cotton leaf worm, *Spodoptera littoralis*. Journal of Insect Physiology. 1974; 20: 1005–14. 10.1016/0022-1910(74)90142-5 4839337

[10] Hegde MG, Santoshakumara MA, Yenagi BS, Patil RK. Influence of weather parameters on pheromone trap catches of *Spodoptera litura* male moth. Journal of Experimental Zoology India. 2016; 19: 1173–76.

[11] Punithavalli M, Sharma AN, Rajkumar MB. Seasonality of the common cutworm *Spodoptera litura* in a soybean ecosystem. Phytoparasitica. 2014; 42: 213–222. 10.1007/s12600-013-0354-5

[12] Kitamura C, KobayashiI M. A comparison between communication disruption and mass trapping methods in mating suppression effect of a synthetic sex pheromone to *Spodoptera litura* F. (Lepidoptera: Noctuidae). Applied Entomology and Zoology. 1985; 20: 222–24. 10.1303/aez.20.222

[13] Carde RT, Minks AK. Control of moth pests by mating disruption: successes and constraints. Annual Review of Entomology. 1995; 40: 559–585. 10.1146/annurev.ento.40.1.559

[14] Srinivas K, Rao P. Management of *Spodoptera litura* (F.) infesting groundnut by mating disruption technique with synthetic sex pheromone. Journal of Entomology Research. 1999; 23: 115–19.

[15] Athanassiou CG, Kavallieratos NG, Mazomenos BE. Effect of trap type, trap color, trapping location, and pheromone dispenser on captures of male *Palpita unionalis* (Lepidoptera: Pyralidae). Journal of Economic Entomology. 2004; 97: 321–29. 10.1603/0022-0493-97.2.321 15154451

[16] Gordon D, Zahavi T, Anshelevich L, Harel M, Ovadia S, Dunkelblum E, et al Mating disruption of *Lobesia botrana* (Lepidoptera: Tortricidae): Effect of pheromone formulations and concentrations. Journal of Economic Entomology. 2005; 98:135–142. 10.1603/0022-0493-98.1.135 15765675

[17] Li QY, Liu JL, Zhao LL, Ma RY. Applications of slow release technique of sex pheromone in pest control. Chinese Journal of Biology Control. 2012; 28:589–593.

[18] Manjili H K, Malvandi H, Mousavi M S, Attari E, Danafar H. In vitro and in vivo delivery of artemisinin loaded PCL–PEG–PCL micelles and its pharmacokinetic study. Artificial cells, nanomedicine, and biotechnology. 2018; 46(5), 926–936. 10.1080/21691401.2017.1347880 28683649

[19] Zamani M, Rostamizadeh K, Manjili H K, Danafar H. In vitro and in vivo biocompatibility study of folate-lysine-PEG-PCL as nanocarrier for targeted breast cancer drug delivery. European Polymer Journal. 2018; 103, 260–270. 10.1016/j.eurpolymj.2018.04.020

[20] Ragaei M, Sabry AKH. Nanotechnology for insect pest control. International Journal of Science Environment Technology.2014;3:528–545.

[21] Stadler T, Buteler M, Valdez SR, Gitto JG. Particulate Nanoinsecticides: A New Concept in Insect Pest Management.2018; 83–108. 10.5772/intechopen.72448

[22] Krober H, Teipel U. Microencapsulation of particles using supercritical carbon dioxide. Chemical Engineering and Processing. 2005; 44:215–19. 10.1016/j.cep.2004.02.014

[23] Patra D, Sanyal A, Rotello VM. Colloidal microcapsules: self-assembly of nanoparticles at the liquid-liquid interface. Chemistry-An Asian Journal. 2010; 5:2442–53. 10.1002/asia.201000301 20936663

[24] Chen Z, Fang Y, Zhang Z. Synthesis and assessment of attractiveness and mating disruption efficacy of sex pheromone microcapsules for the diamondback moth, *Plutellaxy lostella* (L.). Chinese Science Bulletin. 2007; 52(10):1365–1371. 10.1007/s11434-007-0209-x

[25] Wins-Purdy AH, Judd GJR, Evenden ML. Mechanisms of pheromone communication disruption in *Choristoneura rosaceana* exposed to microencapsulated (Z)-11-tetradecenyl acetate formulated with and without horticultural oil. Journal of Chemical Ecology. 2008; 34: 1096–1106. 10.1007/s10886-008-9500-9 18584258

[26] Light DM, Beck JJ. Characterization of microencapsulated pear ester, (2E, 4Z)-Ethyl-2, 4-decadienoate, a kairomonal spray adjuvant against neonate codling moth larvae. Journal of Agriculture Food Chemistry. 2010; 58:7838–45. 10.1021/jf101167p 20527813

[27] Cocco A, Deliperi S, Delrio G. Control of *Tuta absoluta* (Meyrick) (Lepidoptera: Gelechiidae) in greenhouse tomato crops using the mating disruption technique. Journal of Applied Entomology. 2013; 137:16–28. 10.1111/j.1439-0418.2012.01735.x

[28] Stelinski LL, McGhee P, Haas M, Il’ichev AL, Gut LJ. Sprayable microencapsulated sex pheromone formulations for mating disruption of four tortricid species: effects of application height, rate, frequency, and sticker adjuvant. Journal of Economic Entomology. 2007; 100: 1360–69. 10.1603/0022-0493(2007)100[1360:SMSPFF]2.0.CO;2 17849890

[29] Danafar H, Davaran S, Rostamizadeh K, Valizadeh H, Hamidi M. Biodegradable mPEG/PCL core-shell micelles: preparation and characterization as a sustained release formulation for curcumin. Advanced pharmaceutical bulletin. 2014,4(Suppl 2):501–510. 10.5681/apb.2014.074 25671181 PMC4312397

[30] Gharebaghi F, Dalali N, Ahmadi E, Danafar H. Preparation of wormlike polymeric nanoparticles coated with silica for delivery of methotrexate and evaluation of anticancer activity against MCF7 cells. Journal of biomaterials applications.2017,31(9):1305–1316. 10.1177/0885328217698063 28447548

[31] Alami-Milani M, Zakeri-Milani P, Valizadeh H, Salehi R, Salatin S, Naderinia A, et al Novel pentablock copolymers as thermosensitive self-assembling micelles for ocular drug delivery. Advanced pharmaceutical bulletin, 2017,7(1):11 https://doi.org/10.15171/apb.2017.003 28507933 10.15171/apb.2017.003 PMC5426723

[32] Danafar H. Applications of copolymeric nanoparticles in drug delivery systems. Drug research. 2016; 66(10), 506–519. 10.1055/s-0042-109865 27403578

[33] Manjili HRK, Malvandi H, Mousavi MS, Danafar H. Preparation and physicochemical haracterization of biodegradable mPEG-PCL core-shell micelles for delivery of artemisinin. Pharmaceutical Sciences.2016; 22: 234–243. https://doi.org/10.15171/PS.2016.37

[34] Bromberg L. Polymeric micelles in oral chemotherapy. Journal of Control Release. 2008; 128(2): 99–112. 10.1016/j.jconrel.2008.01.018 18325619

[35] Chang TMS. Recent and future developments in modified hemoglobin and microencapsulated hemoglobin as red blood cell substitutes. Artificial Cells, Blood Substitutes and Biotechnology. 1997; 25: 1–24. 10.3109/10731199709118893 9083622

[36] Shulkin A, Stöver HDH. Polymer microcapsules by interfacial polyaddition between styrene-maleic anhydride copolymers and amines. Journal of Membrane Science. 2002; 209: 421–432. 10.1016/S0376-7388(02)00348-4

[37] Lu H, Chen C, Guo H, Zhou X, Dong J, Hong X, et al Determination of the second critical micelle concentration of CTAB by UV spectra without probe. Acta Chimica Sinica-Chinese Edition-, 2006; 64(24): 2437–2441.

[38] Wei XW, Gong CY, Shi S, Fu SZ, Men K, Zeng S, et al Self-assembled honokiol-loaded micelles based on poly (ɛ-caprolactone)-poly (ethylene glycol)-poly (ɛ-caprolactone) copolymer. International Journal of Pharmaceutics. 2009; 369: 170–75. 10.1016/j.ijpharm.2008.10.027 19028556

[39] Abdelbary A, El-Gazayerly ON, El-Gendy NA, Ali AA. Floating tablet of trimetazidine dihydrochloride: an approach for extended release with zero-order kinetics. Aaps Pharmscitech. 2010; 11:1058–67. 10.1208/s12249-010-9468-y 20582493 PMC2974115

[40] Noyes AA, Whitney WR. The rate of solution of solid substances in their own solutions. Journal of the American Chemical Society. 1897; 19: 930–934. 10.1021/ja02086a003

[41] Higuchi T. Mechanism of sustained-action medication. Theoretical analysis of rate of release of solid drugs dispersed in solid matrices. Journal of pharmaceutical sciences. 1963; 52: 1145–9. 10.1002/jps.2600521210 14088963

[42] Hixson AW, Crowell JH. Dependence of reaction velocity upon surface and agitation. Industrial & Engineering Chemistry. 1931; 23(10):1160–1168. 10.1021/ie50262a025

[43] Korsmeyer RW, Gurny R, Doelker EM, Buri P, Peppas NA. Mechanism of solute release from porous hydrophilic polymers. International journal of pharmaceutics. 1983; 15(1): 25–35. 10.1016/0378-5173(83)90064-9

[44] Andreani T, Fangueiro JF, Jose S, Santini MA, Silva A, Souto EB. Hydrophilic polymers for modified-release nanoparticles: a review of mathematical modelling for pharmacokinetic analysis. Current pharmaceutical design. 2015; 21(22): 3090–3096. 10.2174/1381612821666150531163617 26027576

[45] Rostamizadeh K, Manafi M, Nosrati H, Manjili H K, DanafarH. Methotrexate-conjugated mPEG-PCL copolymers: a novel approach for dual triggered drug delivery. New Journal of Chemistry. 2018; 42(8), 5937–5945. 10.1039/C7NJ04864E

[46] Cork A, De Souza K, Hall D R, Jones O T, Casagrande E, Krishnaiah K, Syed Z. Development of PVC-resin-controlled release formulation for pheromones and use in mating disruption of yellow rice stem borer, *Scirpophaga incertulas*. Crop protection. 2008; 27(2): 248–255. 10.1016/j.cropro.2007.05.011

[47] Danafar H, Rostamizadeh K, Davaran S, Hamidi M. Co-delivery of hydrophilic and hydrophobic drugs by micelles: a new approach using drug conjugated PEG-PCL nanoparticles. Drug development and industrial pharmacy.2017; 43(11): 1908–1918. 10.1080/03639045.2017.1355922 28737462

[48] Danafar H, Sharafi A, KheiriManjili H, Andalib S. Sulforaphane delivery using mPEG-PCL co-polymer nanoparticles to breast cancer cells. Pharmaceutical Development and Technology. 2017; 22(5):642–651. 10.3109/10837450.2016.1146296 26916923

[49] Tomaszewska E, Hebert VR, Brunner JF, Jones VP, Doerr M, Hilton R. Evaluation of pheromone release from commercial mating disruption dispensers. Journal of Agricultural Food Chemistry. 2005; 53, 2399–2405. 10.1021/jf048163k 15796569

