# Supplementary figures and images for: Preparation, characterisation, and controlled release of sex pheromone-loaded MPEG-PCL diblock copolymer micelles for Spodoptera litura (Lepidoptera: Noctuidae)
Source: PLoS One. 2018 Sep 7;13(9):e0203062. doi: 10.1371/journal.pone.0203062 (PMC6128524; doi:10.1371/journal.pone.0203062)

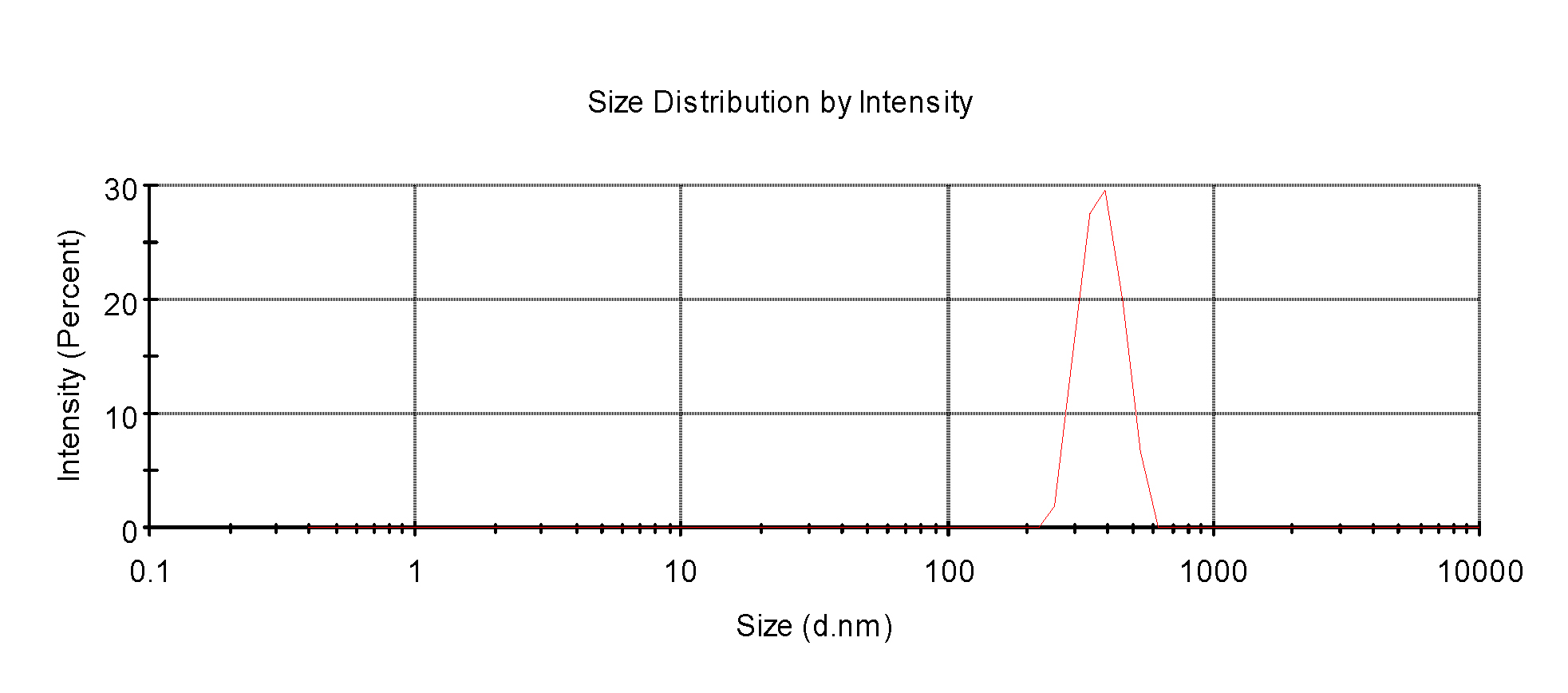

Supplement: S1 Fig — (TIF) [file pone.0203062.s001.tif]

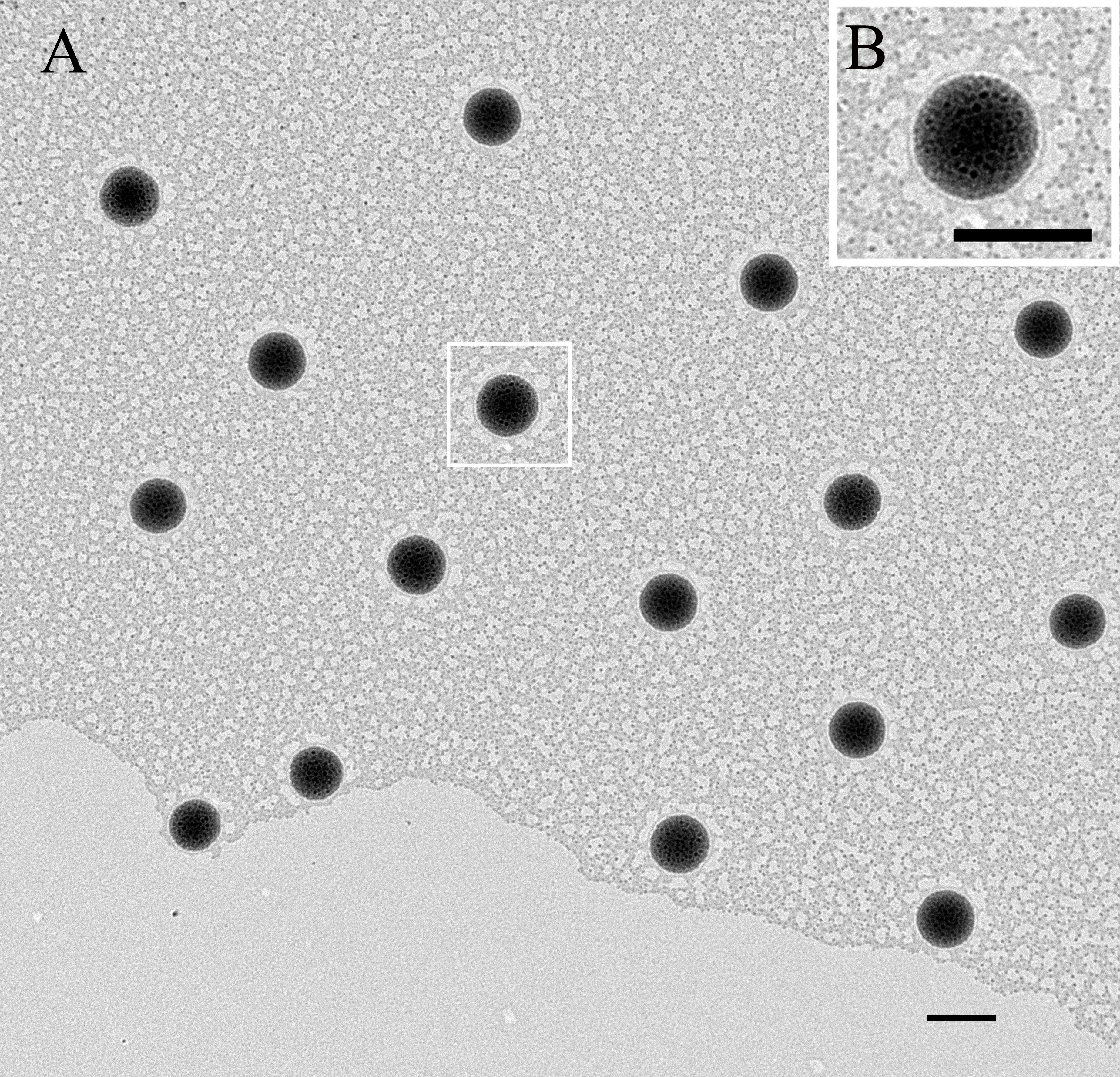

Supplement: S2 Fig — Bars in panel A and panel B are 400 nm. Panel B is an enlarged view of the boxed area in Panel A. (TIF) [file pone.0203062.s002.tif]

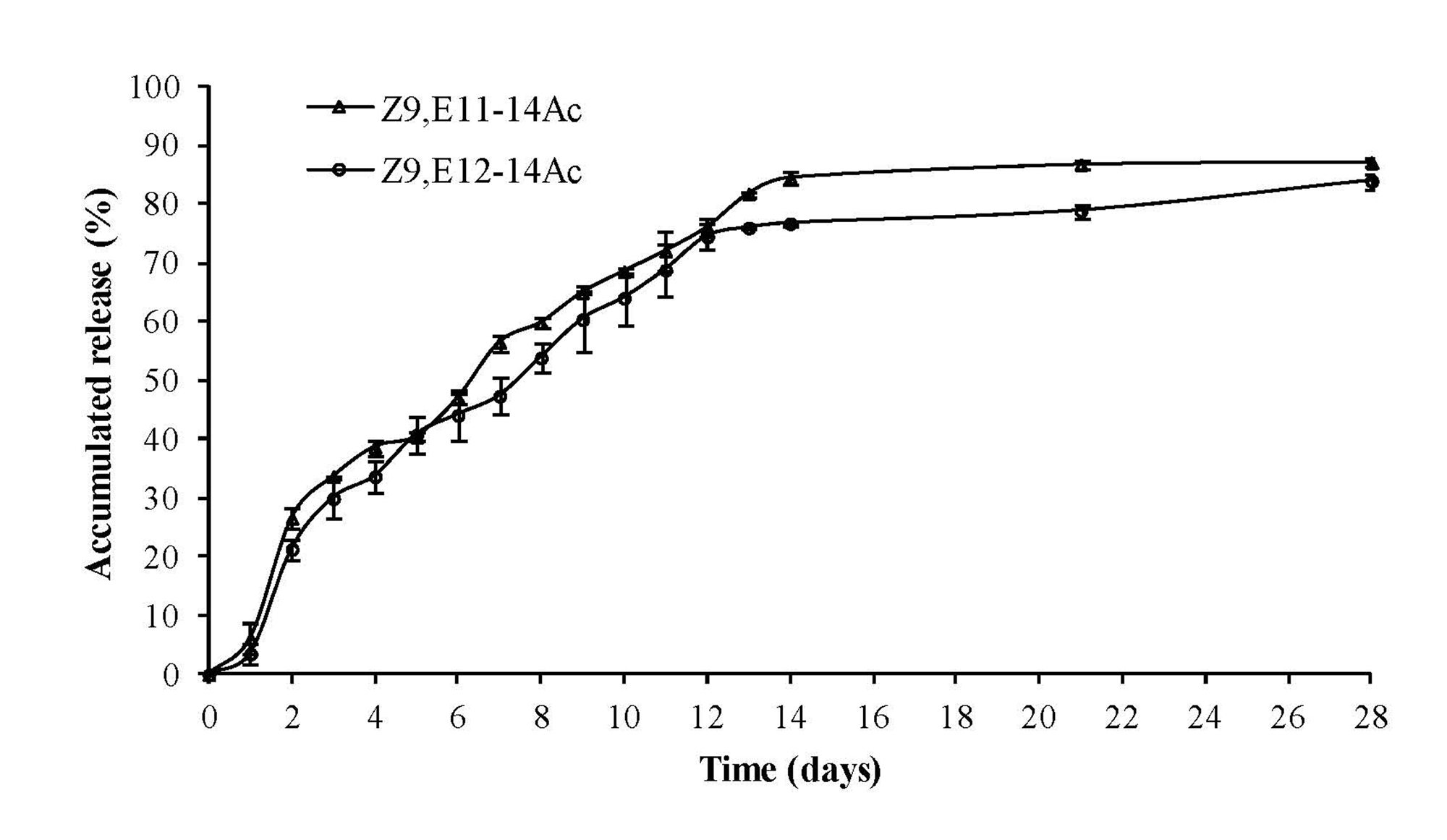

Supplement: S3 Fig — The S. litura sex pheromone has two components, Z9,E11-14:Ac and Z9,E12-14:Ac. Data are expressed as means ± S.D. (n = 3). (TIF) [file pone.0203062.s003.tif]

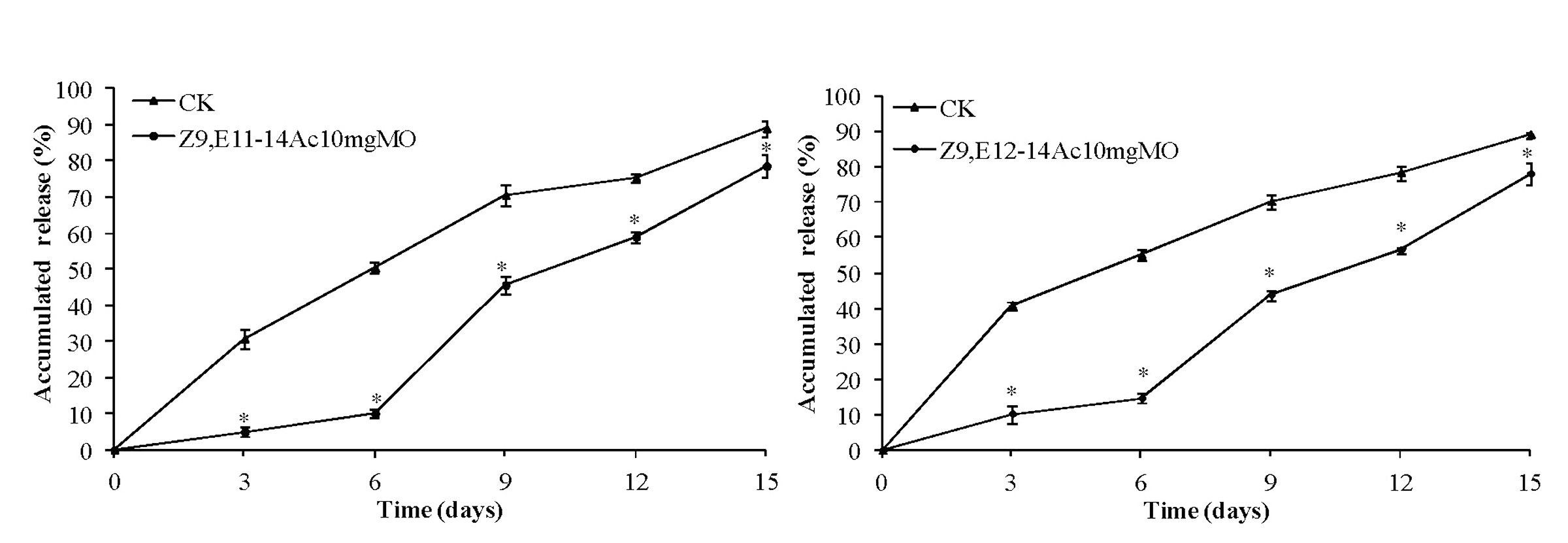

Supplement: S4 Fig — Data are expressed as means ± S.D. (n = 3). Asterisks (*) indicate significance (P ≤ 0.05). (TIF) [file pone.0203062.s004.tif]
