# Supplementary material for: Preparation, characterisation, and controlled release of sex pheromone-loaded MPEG-PCL diblock copolymer micelles for Spodoptera litura (Lepidoptera: Noctuidae)
Source: PLoS One. 2018 Sep 7;13(9):e0203062. doi: 10.1371/journal.pone.0203062 (PMC6128524; doi:10.1371/journal.pone.0203062)
Supplement: S1 Table — W (wall-forming materials), W/S ratio (the mass ratio of sex pheromone to wall-forming materials), T (reaction temperature), S (stirring speed). (DOC) [file pone.0203062.s005.doc]

Table 1. Experimental factors and their levels in orthogonal projects

| **Level** | **W**  **(A)** | **W/S ratio (w/w)**  **(B)** | **T (°C)**  **(C)** | **S (rpm)**  **(D)** |
| --- | --- | --- | --- | --- |
| **1** | MPEG_5000_-PCL_2000_ | 2:1 | 30 | 800 |
| **2** | MPEG_3000_-PCL_2300_ | 2.5:1 | 40 | 1000 |
| **3** | MPEG_5000_-PCL_10000_ | 5:1 | 50 | 1200 |

W (wall-forming materials), W/S ratio (the mass ratio of sex pheromone to wall-forming materials), T (reaction temperature), S (stirring speed).
