# Supplementary material for: Preparation, characterisation, and controlled release of sex pheromone-loaded MPEG-PCL diblock copolymer micelles for Spodoptera litura (Lepidoptera: Noctuidae)
Source: PLoS One. 2018 Sep 7;13(9):e0203062. doi: 10.1371/journal.pone.0203062 (PMC6128524; doi:10.1371/journal.pone.0203062)
Supplement: S2 Table — W (wall-forming materials), W/S ratio (the mass ratio of sex pheromone to wall-forming materials), T (reaction temperature), S (stirring speed), EE (encapsulation efficiency of micelle). The arrangements of A, B, C, D were decided by orthogonal design for 4 (factor) × 9 (run number). (DOC) [file pone.0203062.s006.doc]

**Table 2. Results of the L_9_(3^4^) orthogonal experiment using Z9,E11-14:Ac MPEG-PCL nanoparticles**

| **Factor** | **W**  **(A)** | **W/S ratio (w/w)**  **(B)** | **T (°C)**  **(C)** | **S (rpm)**  **(D)** | **EE (%)**  **(Z9:E11-14:Ac)%** |
| --- | --- | --- | --- | --- | --- |
| **1** | 1 | 1 | 1 | 1 | 76.76 |
| **2** | 1 | 2 | 2 | 2 | 76.51 |
| **3** | 1 | 3 | 3 | 3 | 51.10 |
| **4** | 2 | 1 | 2 | 3 | 53.95 |
| **5** | 2 | 2 | 3 | 1 | 65.45 |
| **6** | 2 | 3 | 1 | 2 | 78.04 |
| **7** | 3 | 1 | 3 | 2 | 61.26 |
| **8** | 3 | 2 | 1 | 3 | 55.77 |
| **9** | 3 | 3 | 2 | 1 | 60.74 |
| **K_1_** | 204.37 | 191.96 | 210.57 | 202.95 |  |
| **K_2_** | 197.44 | 197.73 | 191.20 | 215.81 |  |
| **K_3_** | 177.77 | 189.89 | 177.80 | 160.82 |  |
| **k_1_** | 68.12 | 63.99 | 70.19 | 67.65 |  |
| **k_2_** | 65.81 | 65.91 | 63.73 | 71.94 |  |
| **k_3_** | 59.26 | 63.30 | 59.27 | 53.61 |  |
| **R** | 8.87 | 2.61 | 10.92 | 18.33 |  |
| **Influence degree of factors** | S > T > W > W/S ratios | | | | |
| **Best group** | A_1_B_2_C_1_D_2_  W (MPEG_5000_-PCL_2000_) - W/S ratio (2.5:1) - T (30°C) - S (1000 rpm) | | | | |

W (wall-forming materials), W/S ratio (the mass ratio of sex pheromone to wall-forming materials), T (reaction temperature), S (stirring speed), EE (encapsulation efficiency of micelle). The arrangements of A, B, C, D were decided by orthogonal design for 4 (factor) × 9 (run number).
