# Supplementary material for: Preparation, characterisation, and controlled release of sex pheromone-loaded MPEG-PCL diblock copolymer micelles for Spodoptera litura (Lepidoptera: Noctuidae)
Source: PLoS One. 2018 Sep 7;13(9):e0203062. doi: 10.1371/journal.pone.0203062 (PMC6128524; doi:10.1371/journal.pone.0203062)
Supplement: S3 Table — W (wall-forming materials), W/S ratio (the mass ratio of sex pheromone to wall-forming materials), T (reaction temperature), S (stirring speed), and EE (encapsulation efficiency of micelle). The arrangements of A, B, C, D were decided by orthogonal design for 4 (factor) × 9 (run number). (DOC) [file pone.0203062.s007.doc]

**Table 3. Results of the L_9_(3^4^) orthogonal experiment using Z9,E12-14:Ac MPEG-PCL nanoparticles**

| **Factor** | **W**  **(A)** | **W/S ratio (w/w)**  **(B)** | **T (°C)**  **(C)** | **S (rpm)**  **(D)** | **EE (%)**  **(Z9:E12-14:Ac)%** |
| --- | --- | --- | --- | --- | --- |
| **1** | 1 | 1 | 1 | 1 | 77.11 |
| **2** | 1 | 2 | 2 | 2 | 85.77 |
| **3** | 1 | 3 | 3 | 3 | 58.91 |
| **4** | 2 | 1 | 2 | 3 | 55.41 |
| **5** | 2 | 2 | 3 | 1 | 71.66 |
| **6** | 2 | 3 | 1 | 2 | 82.73 |
| **7** | 3 | 1 | 3 | 2 | 60.23 |
| **8** | 3 | 2 | 1 | 3 | 56.45 |
| **9** | 3 | 3 | 2 | 1 | 57.95 |
| **K_1_** | 221.79 | 192.74 | 216.29 | 206.72 |  |
| **K_2_** | 209.79 | 213.88 | 199.13 | 228.73 |  |
| **K_3_** | 174.63 | 199.59 | 190.80 | 170.77 |  |
| **k_1_** | 73.93 | 64.25 | 72.10 | 68.91 |  |
| **k_2_** | 69.93 | 71.29 | 66.38 | 76.24 |  |
| **k_3_** | 58.21 | 66.53 | 63.60 | 56.92 |  |
| **R** | 15.72 | 7.05 | 8.50 | 19.32 |  |
| **Influence degree of factors** | S > W > T > W/S ratios | | | | |
| **Best group** | A_1_B_2_C_1_D_2_  W (MPEG_5000_-PCL_2000_) - W/S ratio (2.5:1) - T (30°C) - S (1000 rpm) | | | | |

W (wall-forming materials), W/S ratio (the mass ratio of sex pheromone to wall-forming materials), T (reaction temperature), S (stirring speed), and EE (encapsulation efficiency of micelle). The arrangements of A, B, C, D were decided by orthogonal design for 4 (factor) × 9 (run number).
