# Supplementary material for: Preparation, characterisation, and controlled release of sex pheromone-loaded MPEG-PCL diblock copolymer micelles for Spodoptera litura (Lepidoptera: Noctuidae)
Source: PLoS One. 2018 Sep 7;13(9):e0203062. doi: 10.1371/journal.pone.0203062 (PMC6128524; doi:10.1371/journal.pone.0203062)
Supplement: S4 Table — SS (Sum of square), df (degree of freedom), MS (mean square), F (critical value). ‘*’and ‘**’ represent significant difference (P ≤ 0.05) and extremely significant difference (P ≤ 0.01), respectively. (DOC) [file pone.0203062.s008.doc]

**Table 4. Analysis of the orthogonal experiment results of Z9,E11-14:Ac MPEG-PCL nanoparticles using ANOVA**

| **Source of variation** | ***SS*** | ***df*** | ***MS*** | **F** |
| --- | --- | --- | --- | --- |
| **A** | 380.931 | 2 | 190.466 | 7.554** |
| **B** | 33.001 | 2 | 16.501 | 6.540* |
| **C** | 542.744 | 2 | 271.372 | 10.763** |
| **D** | 1655.042 | 2 | 827.521 | 32.821** |
| **error** | 453.842 | 18 | 25.213 |  |

*SS* (Sum of square), *df* (degree of freedom), *MS* (mean square), F (critical value). ‘*’and ‘**’represent significant difference (*P* ≤ 0.05) and extremely significant difference (*P* ≤ 0.01), respectively.
