# Supplementary material for: Preparation, characterisation, and controlled release of sex pheromone-loaded MPEG-PCL diblock copolymer micelles for Spodoptera litura (Lepidoptera: Noctuidae)
Source: PLoS One. 2018 Sep 7;13(9):e0203062. doi: 10.1371/journal.pone.0203062 (PMC6128524; doi:10.1371/journal.pone.0203062)
Supplement: S5 Table — SS (Sum of square), df (degree of freedom), MS (mean square), F (critical value). ‘*’and ‘**’ represent significant difference (P ≤ 0.05) and extremely significant difference (P ≤ 0.01), respectively. (DOC) [file pone.0203062.s009.doc]

**Table 5. Analysis of the orthogonal experiment results of Z9,E12-14:Ac MPEG-PCL nanoparticles using ANOVA**

| **Source of variation** | ***SS*** | ***df*** | ***MS*** | **F** |
| --- | --- | --- | --- | --- |
| **A** | 121.833 | 2 | 600.917 | 20.087** |
| **B** | 232.648 | 2 | 116.324 | 3.888* |
| **C** | 337.751 | 2 | 168.875 | 5.645* |
| **D** | 1711.861 | 2 | 855.930 | 28.611** |
| **Consolidated error** | 538.493 | 18 | 29.916 |  |

*SS* (Sum of square), *df* (degree of freedom), *MS* (mean square), F (critical value). ‘*’and ‘**’represent significant difference (*P* ≤ 0.05) and extremely significant difference (*P* ≤ 0.01), respectively.
