# Supplementary material for: Preparation, characterisation, and controlled release of sex pheromone-loaded MPEG-PCL diblock copolymer micelles for Spodoptera litura (Lepidoptera: Noctuidae)
Source: PLoS One. 2018 Sep 7;13(9):e0203062. doi: 10.1371/journal.pone.0203062 (PMC6128524; doi:10.1371/journal.pone.0203062)
Supplement: S6 Table — EE (encapsulation efficiency of micelle), A1B2C1D2 [best group: W (MPEG5000-PCL2000)—W/S ratio (2.5:1)—T (30°C)—S (1000 rpm)]. (DOC) [file pone.0203062.s010.doc]

**Table 6. Test of verification**

| **Best group** | **EE (%)** | |
| --- | --- | --- |
|  | **Z9,E11-14:Ac** | **Z9,E12-14:Ac** |
| **A_1_B_2_C_1_D_2_** | 82.91 | 88.73 |
| **A_1_B_2_C_1_D_2_** | 84.54 | 87.84 |
| **A_1_B_2_C_1_D_2_** | 82.21 | 89.32 |
| **Mean ± SE** | 83.22 ± 0.35 | 88.63 ± 0.56 |

EE (encapsulation efficiency of micelle), A_1_B_2_C_1_D_2_ [best group: W (MPEG_5000_-PCL_2000_) - W/S ratio (2.5:1) - T (30°C) - S (1000 rpm)]
