# Supplementary material for: Preparation, characterisation, and controlled release of sex pheromone-loaded MPEG-PCL diblock copolymer micelles for Spodoptera litura (Lepidoptera: Noctuidae)
Source: PLoS One. 2018 Sep 7;13(9):e0203062. doi: 10.1371/journal.pone.0203062 (PMC6128524; doi:10.1371/journal.pone.0203062)
Supplement: S7 Table — (DOC) [file pone.0203062.s011.doc]

**Table 7. Stability of micelles during storage period**

| **Store temperature (**°C**)** | **Mean size of micelles immediately after preparation (nm)** | **Mean size of micelles after 15 day (nm)** | **Mean size of micelles after 30 day (nm)** |
| --- | --- | --- | --- |
| 2 | 374 | 377 | 386 |
| 4 | 374 | 379 | 391 |
| 8 | 374 | 384 | 402 |
