# Supplementary material for: Preparation, characterisation, and controlled release of sex pheromone-loaded MPEG-PCL diblock copolymer micelles for Spodoptera litura (Lepidoptera: Noctuidae)
Source: PLoS One. 2018 Sep 7;13(9):e0203062. doi: 10.1371/journal.pone.0203062 (PMC6128524; doi:10.1371/journal.pone.0203062)
Supplement: S8 Table — (DOC) [file pone.0203062.s012.doc]

**Table 8. Results of release kinetics model fitting of MPEG_5000_-PCL_2000_** micelles

| **Model** | **Z9,E11-14:Ac** | | | **Z9,E12-14:Ac** | | |
| --- | --- | --- | --- | --- | --- | --- |
|  | **Intercept** | **Slope** | **R^2^** | **Intercept** | **Slope** | **R^2^** |
| **Zero-order model** | 25.5268 | 3.2234 | 0.8599 | 22.2329 | 3.1157 | 0.8670 |
| **First-order model** | 0.2101 | 0.0853 | 0.9598 | 0.1886 | 0.0721 | 0.9608 |
| **Higuchi model** | 1.0032 | -0.2014 | 0.9364 | 1.0237 | -0.1932 | 0.9398 |
| **Korsmeyer-Peppas model** | 0.1221 | 0.69(n) | 0.9298 | 0.0952 | 0.58(n) | 0.9339 |
| **Hixson-Crowell model** | 0.4441 | -0.0230 | 0.6861 | 0.4736 | -0.0236 | 0.6987 |
