# Supplementary material for: Preparation, characterisation, and controlled release of sex pheromone-loaded MPEG-PCL diblock copolymer micelles for Spodoptera litura (Lepidoptera: Noctuidae)
Source: PLoS One. 2018 Sep 7;13(9):e0203062. doi: 10.1371/journal.pone.0203062 (PMC6128524; doi:10.1371/journal.pone.0203062)
Supplement: S9 Table — The first-order kinetic model: lnCt = lnC0 +K1·t, R2 = regression coefficient. (DOC) [file pone.0203062.s013.doc]

**Table 9. Influence of a controlled-release agent on the controlled-release performance of sex pheromone-loaded MPEG_5000_-PCL_2000_ micelles**

| **Compound** | **Mass of MO (mg/mL)** | **Half-life (days)** | **First**-**order release parameters^a^** | | |
| --- | --- | --- | --- | --- | --- |
|  |  |  | **Slope** | **Intercept** | **R^2^** |
| **Z9,E11-14:Ac** | CK | 5.4 | -0.1395 | 0.0636 | 0.9866 |
|  | 10 | 9.1 | -0.1021 | 0.2326 | 0.9432 |
|  | 20 | 8.7 | -0.1064 | 0.2284 | 0.9485 |
|  | 30 | 8.6 | -0.1086 | 0.2377 | 0.9475 |
| **Z9,E12-14:Ac** | CK | 5.0 | -0.1374 | -0.0127 | 0.9919 |
|  | 10 | 9.2 | -0.0967 | 0.1935 | 0.9432 |
|  | 20 | 8.9 | -0.0998 | 0.1988 | 0.9435 |
|  | 30 | 8.7 | -0.1032 | 0.2064 | 0.9428 |

^a^The first-order kinetic model: lnC_t_ =lnC_0_ +K_1_·t, R^2^ = regression coefficient.
